# Prospective evaluation of microscopic extension using whole-mount preparation in patients with hepatocellular carcinoma: Definition of clinical target volume for radiotherapy

**DOI:** 10.1186/1748-717X-5-73

**Published:** 2010-08-23

**Authors:** Weihu Wang, Xiaoli Feng, Tao Zhang, Jing Jin, Shulian Wang, Yueping Liu, Yongwen Song, Xinfan Liu, Zihao Yu, Yexiong Li

**Affiliations:** 1 Department of Radiation Oncology, Cancer Hospital, Chinese Academy of Medical Sciences (CAMS) and Peking Union Medical College (PUMC), Beijing 100021, P.R. China; 2 Department of Pathology, Cancer Hospital, Chinese Academy of Medical Sciences (CAMS) and Peking Union Medical College (PUMC), Beijing 100021, P.R. China

## Abstract

**Background:**

To define the clinical target volume (CTV) for radiotherapy in patients with hepatocellular carcinoma (HCC).

**Methods:**

A prospective study was conducted to histologically evaluate the presence and the distance of microscopic extension (ME) for resected HCC on the basis of examination of whole-mount preparations of carcinoma tissue sections.

**Results:**

A total of 380 whole-mount slides prepared from tumor samples of 76 patients with HCC were examined. Patients with elevated pretreatment AFP levels exhibited higher risk of ME as compared to those with normal pretreatment AFP levels (93.9% vs. 69.8%, *P *< 0.01). ME positivity was 16.7% for Grade 1, 79.1% for Grade 2, and 96.3% for Grade 3 tumors (*P *< 0.01). The mean distance of ME was 0.0 ± 0.1 mm (range 0-0.2 mm) for Grade 1, 0.9 ± 0.9 mm (range 0-4.5 mm) for Grade 2, and 1.9 ± 1.9 mm (range 0-8.0 mm) for Grade 3 tumors (*P *< 0.01).

**Conclusions:**

The CTV margins for tumor Grades 1, 2, and 3 HCC, are recommended to be 0.2 mm, 4.5 mm, and 8.0 mm beyond the gross tumor margin, respectively, to account for possible ME of the tumors in all patients.

## Background

Hepatocellular carcinoma (HCC) is the fifth most common malignancy and the third leading cause of cancer mortality worldwide [1]. It is estimated that in 2009, there will be 22,620 new cases of liver cancer and 18,160 related deaths in America [2]. It is the third most common cancer and the second leading cause of cancer-related death in China [3,4]. The mainstay of therapy is surgical resection with a 5-year survival rate ranging from 30 to 70% [5,6]. Unfortunately, less than 20% of HCC patients are eligible for surgery; surgery is ruled out in many patients because of inadequate liver function reserve, the multifocal nature of the disease, and the proximity to and/or involvement of vascular or biliary structures [7,8]. Traditionally, the role of radiotherapy in the management of HCC has been limited by the low tolerance of the liver to radiation. However, recent advances in radiation techniques, such as three-dimensional conformal radiation therapy (3D-CRT), intensity-modulated radiation therapy (IMRT), image-guided radiation therapy (IGRT), proton therapy, tumor-tracking, and respiratory gating techniques, have allowed the administration of high radiation doses to the primary tumor with sparing of the normal liver tissue [9,10]. Thus, the use of radical radiotherapy for unresectable HCC has increased dramatically in recent years, and promising results have been achieved [11-13]. In a radiotherapy setting, a tissue volume inclusive of the subclinical lesions in addition to the gross tumor is defined as clinical target volume (CTV). Modern imaging techniques enable precise delineation of gross tumor volume (GTV); however, none of the available imaging techniques enable the detection of the actual distance of the microscopic extension (ME) of HCC. It has not been possible to clearly define the CTV of HCC. Taking ME into account, a margin of 1.0 to 1.5 cm is arbitrarily added to the GTV to obtain the CTV [11-13]. It remains to be ascertained whether this margin is adequate to cover ME in patients with HCC. A narrow margin is associated with the increased risk of local failure, while a generous margin results in increased radiation damage to normal tissues. Therefore, guidelines for the extent of ME to be included within the radiation volume would be very useful in clinical practice. In this prospective study, we histologically evaluated the ME of HCC using whole-mount slides that should allow for a more representative assessment of the ME by increasing the amount of tissue examined compared to routine small histopathologic slides and defined the CTV as precisely as possible.

## Materials

### Case selection

Between June, 2007, and March, 2009, we prospectively enrolled 76 patients with pathologically diagnosed HCC who underwent tumor resection at the Cancer Institute and Hospital, Peking Union Medical College and Chinese Academy of Medical Sciences (Beijing, China). The pretreatment evaluation for all patients consisted of a complete history taking, physical examination, serological tests to screen for hepatitis B virus (HBV) and hepatitis C virus (HCV), α-fetoprotein (AFP) assay, prothrombin time test (PT), complete blood counts, serum biochemical tests, abdominal computed tomography (CT) with arterial- and portal-venous-phase imaging or MRI, and chest radiography. The time scale from imaging studies and laboratory tests to surgery was within 3 weeks before surgery. The criteria for inclusion in this study were as follows: (1) resectable hepatic lesion with clinical stage I and II disease determined according to the American Joint Committee on Cancer (AJCC 2002); (2) radical resection with a margin ≥1 cm around the gross tumor; (3) detection of a single lesion without satellite nodules in the pretreatment CT or during the operation; (4) no history of previous treatment. The protocol was approved by the institutional review boards, and written informed consent was obtained from all the patients.

### ME measurement

After fixation of the resected specimen in 13% neutral buffered formalin for ≥12 h, transverse sections were obtained with an average thickness of 10 mm, which were completely embedded in paraffin blocks. The paraffin blocks were cut into 4-μm-thick sections, and whole mount slides of these tissues were stained with hematoxylin and eosin (HE) and evaluated using a light microscope (Olympus BX40; Olympus, Tokyo, Japan). To avoid interobserver variations, the same pathologist (Xiaoli Feng) who particularly experienced in the assessment of hepatic lesions assessed all the slides and identified evidence of ME. ME was considered as positive if extension was detected microscopically, even if it was absent on imaging studies. If a tumor had multiple MEs, the longest distance between the tumor margin of the ME was recorded. ME was determined by light microscopy at a magnification of ×40 and was confirmed, if required, by examination at ×100 magnification.

The following features were recorded for each case: (1) ME status (positive versus negative), defined as positive if ME was identified; (2) ME distance, defined as the maximum linear distance from the capsular margin of the primary gross tumor to the farthest extent of the ME. We did not account for tissue shrinkage in this study because of the fixation of the tumor sections.

### Statistical analysis

Statistical analysis was performed using SPSS version 11.0 statistical package (SPSS Inc., Chicago, IL). The clinical characteristics were analyzed using a chi-square test or Fisher's exact test (two-tailed) for categorical variables and logistic regression analysis for continuous variables. The Student's unpaired *t *test was used to determine the significance of the difference between the 2 sample means. The association of these characteristics with ME distance was analyzed using logistic or linear regression models. Multivariate analyses were performed using a multiple logistic regression model. A *P *value less than or equal to 0.05 was considered statistically significant.

## Results

### Patient characteristics

Patient characteristics and ME status are summarized in Table 1. The median age at the time of operation was 53 years (range, 25-78 years). The median pretreatment AFP level was 13.1 ng/ml (mean, 1776.9 ng/ml; range, 1~50781 ng/ml). The median tumor size measured on enhanced CT was 5.0 cm (mean, 5.1 cm; range 1.6-10.2 cm) in the greatest dimension. Only 1 of the patients had Child-Pugh Class B cirrhosis, while the others had Child-Pugh Class A cirrhosis.

**Table 1:** Patient characteristics and ME status

	Patients	ME positive	
**Characteristic**	**No (%)**	**%**	***P *value**

Age (y)			NS
≤60	39 (51.3)	87.2	
>60	37 (48.7)	73.0	
Sex			NS
Male	60 (78.9)	78.3	
Female	16 (21.1)	87.5	
Status of Hepatitis			
HBsAg positive	62 (81.6)	85.5	NS
HCVAb positive	3 (3.9)	33.3	
Others	11 (14.5)	63.6	
AFP level (ng/ml)			<0.01
Normal	43 (56.6)	69.8	
Elevated	33 (43.4)	93.9	
Tumor size (cm)			NS
≤5	41 (53.9)	78.0	
>5	35 (46.1)	82.9	
TNM stage			NS
I	42 (55.3)	83.3	
II	34 (44.7)	76.5	
Platelets (G/L)			NS
<100	11 (14.5)	81.8	
≥100	65 (85.5)	80.0	
AST level (U/L)			NS
≤40	54 (71.1)	85.2	
>40	22 (28.9)	68.2	
ALT level (U/L)			NS
≤40	49 (71.1)	81.6	
>40	27 (28.9)	77.8	
GGT level (U/L)			NS
≤55	43 (56.6)	81.4	
>55	33 (43.4)	78.8	
Albumin level(g/L)			NS
≤35	8 (10.5)	75.0	
>35	68 (89.5)	80.9	
BIL (μmol/L)			NS
≤17.1	59 (77.6)	78.0	
>17.1	17 (22.4)	88.2	
PT(s)			NS
≤13.3	55 (72.4)	83.6	
>13.3	21 (27.6)	71.4	
Tumor grade			<0.01
1	6 (7.9)	16.7	
2	43 (56.6)	79.1	
3	27 (35.5)	96.3	
All patients	76 (100)	80.3	--

*Abbreviations: *HBsAg = hepatitis B surface antigen; HCV Ab = hepatitis C virus antibody; AFP = serum α-fetoprotein; AST = serum aspartate aminotransferase level; ALT = serum alanine aminotransferase level; GGT = serum γ-glutamyltransferase; BIL = serum total bilirubin level; PT = prothrombin time; NS = not significant.

### Factors associated with the presence of ME

In this study, the average number of slides for each patient investigated histologically was 5 (range 2-11 slides), and 380 slides of these tumor specimens were reviewed. Of the 76 patients with HCC, 61 (80.3%) patients had ME, and 15 (19.7%) patients did not have ME. As shown in Table 1, factors associated with the presence of ME included pretreatment AFP level and tumor grade. Patients with elevated pretreatment AFP level (>25 ng/ml) exhibited higher risk of ME compared with those with normal pretreatment levels (93.9% vs. 69.8%, *P *< 0.01). ME was more frequently detected in high-grade tumors than in low-grade tumors. ME positivity was 16.7% (1/6) for Grade 1, 79.1% (34/43) for Grade 2, and 96.3% (26/27) for Grade 3 tumors (*P *< 0.01). The pretreatment hepatitis B surface antigen and hepatitis C virus antibody statuses; the serum levels of aspartate aminotransferase, alanine aminotransferase, γ-glutamyltransferase, total bilirubin, and albumin; platelet count; tumor size; tumor-node-metastasis (TNM) stage; and prothrombin time did not correlate with the presence of ME. On multivariate analysis, only tumor grade remained significantly and independently associated with ME positivity (*P *< 0.01).

### Factors associated with ME distance

The ME distance for HCC are listed in Table 2. Of the 76 patients, the median ME distance was 1.0 mm (mean, 1.2 mm; range, 0-8 mm). The majority (72/76, 94.7%) of patients had ME ≤ 3.5 mm, whereas 4 patients had ME of 4.5 mm (n = 2), 7.0 mm and 8.0 mm, respectively. Only the tumor grade was found to significantly correlate with the distance of ME (*P *< 0.01).

**Table 2:** ME distance (mm) by clinical characteristics

Characteristic	ME extent (mean ± SD)	*P *value
Age (y)		NS
≤60	1.3 ± 1.4	
>60	1.0 ± 1.5	
Sex		NS
Male	1.2 ± 1.5	
Female	1.1 ± 0.9	
Status of Hepatitis		
HBsAg positive	1.3 ± 1.5	NS
HCVAb positive	0.3 ± 0.6	
Others	1.0 ± 1.1	
AFP level (ng/ml)		NS
Normal	1.1 ± 1.5	
Elevated	1.3 ± 1.3	
Tumor size (cm)		NS
≤5	1.2 ± 1.3	
>5	1.2 ± 1.5	
TNM stage		NS
I	1.2 ± 1.4	
II	1.2 ± 1.5	
Platelets (G/L)		NS
<100	1.3 ± 2.0	
≥100	1.2 ± 1.3	
AST level (U/L)		NS
≤40	1.3 ± 1.5	
>40	1.0 ± 1.3	
ALT level (U/L)		NS
≤40	1.2 ± 1.4	
>40	1.1 ± 1.5	
GGT level (U/L)		NS
≤55	1.0 ± 0.9	
>55	1.4 ± 1.9	
Albumin level(g/L)		NS
≤35	1.0 ± 0.8	
>35	1.2 ± 1.5	
BIL (μmol/L)		NS
≤17.1	1.2 ± 1.5	
>17.1	1.1 ± 1.1	
PT(s)		NS
≤13.3	1.3 ± 1.5	
>13.3	1.0 ± 1.1	
Tumor grade		<0.01
1	0.0 ± 0.1	
2	0.9 ± 0.9	
3	1.9 ± 1.9	

All patients	1.2 ± 1.4	--

*Abbreviations*: As in table 1.

**Figure 1:**
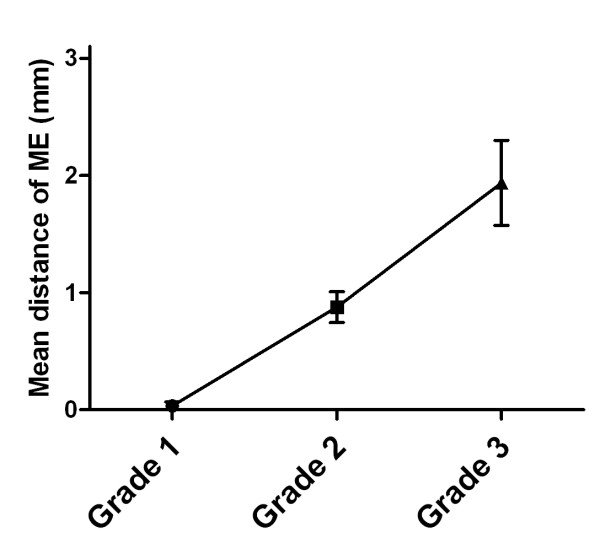
**Correlation between tumor grades and the mean distance of microscopic extension (ME)**.

Of the 76 patients, 6 (7.9%) patients had tumors of Grade 1; 43 (56.6%), of Grade 2; and 27 (35.5%), of Grade 3. Figure 1 shows the relationship between tumor grade and the mean distance of ME, with higher grade tumors having a greater distance of ME. When evaluated by tumor grade, the distance (mean ± SD) of ME was 0.0 ± 0.1 mm (median, 0.0 mm; range 0-0.2 mm) for Grade 1, 0.9 ± 0.9 mm (median, 0.80 mm; range 0-4.5 mm) for Grade 2, and 1.9 ± 1.9 mm (median, 1.5 mm; range 0-8.0 mm) for Grade 3 tumors (*P *< 0.01).

**Figure 2:**
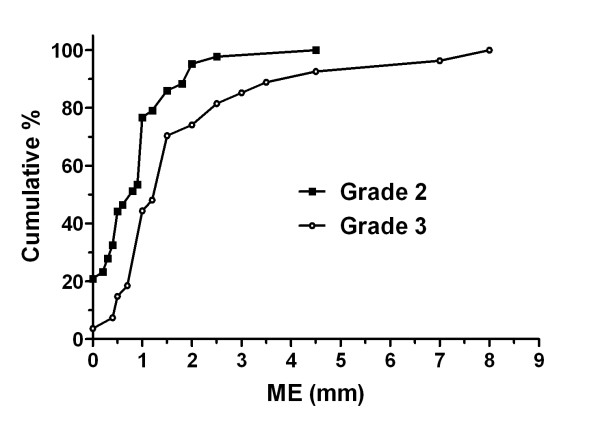
**Cumulative distribution of microscopic extension (ME) according to tumor grade**. Grade 1 was not included in this analysis due to the small number of patients with tumors of this grade, and 5 out of 6 patients did not have ME.

Figure 2 presents the cumulative distribution of the ME in patients with tumors of Grades 2 and 3. A comparison between the distributions of these 2 grades indicated that most of the Grade 2 (67.4%) and Grade 3 (66.7%) tumors had ME of 0.1-1.9 mm. Grade 1 tumors were not included in this analysis owing to the small number of patients with this tumor type, and 5 out of 6 patients did not have ME.

## Discussion

Microscopic extension of the primary tumor plays a significant role in defining the CTV for radiotherapy. A quantitative pathologic assessment of subclinical tumor invasion from primary tumor or metastatic lymph nodes into adjacent tissues for planning external-beam radiotherapy has been performed successfully for some cancers, including head-and-neck, lung, skin, prostate, and bladder cancers [14-22]. There are few relevant data available for hepatocellular carcinoma [23]. To our knowledge, this is the first prospective study to evaluate the ME of tumors using whole-mount preparations in patients with hepatocellular carcinoma. The present study showed that ME occurred more frequently among patients with an elevated AFP level than those with normal AFP levels. The tumor grade is significantly associated with both the presence and the extent of ME.

Developments in radiation techniques have enabled the safe delivery of dose-escalated conformal radiation to a wide spectrum of patients with inoperable HCC. Several studies have shown an improved response and survival rates with the administration of a high radiation dose in these patients [12,24-26]. The University of Michigan conducted a phase I/II trial of 128 patients with unresectable intrahepatic malignancies receiving 3D-CRT and concurrent hepatic arterial infusion of floxuridine. Patients who received a dose ≥75 Gy had significantly higher survival rates compared to those receiving a dose of <75 Gy (23.9 months vs. 14.9 months, *P *< 0.01) [24,27]. Similarly, Park et al. and Seong et al. reported the results of a study on 158 HCC patients classified with Child-Pugh class A or B liver disease, who were prescribed radiation doses of 25-60 Gy in daily fractions of 1.8-Gy [25,28]. The total radiation dose was found to be the only significant factor in predicting tumor response and survival. Other studies have also reported that the radiation dose appeared to be a significant prognostic factor in radiation response of patients with HCC [11,12,26]. However, the treatment benefits observed with increased radiation doses come at a cost because toxicity risks increase proportionally with the increases in radiation dose and large treatment volume [25]. Even in 3D-CRT or IMRT, the dose delivery for the disease is limited by the sensitivity of liver tissue to ionizing radiation. Therefore, in the case of high-dose 3D-CRT or IMRT for HCC treatment, the adequate treatment of the tumor, including the primary tumor and subclinical lesions and the radiation tolerance of the healthy surrounding tissue need to be balanced well. In other words, it is very important to estimate the microscopic extension as accurately as possible. Unfortunately, there was no precise definition of the optimal CTV margins; therefore, no optimal treatment plan for patients with HCC has been defined. Several previous studies have used a margin of 1.0 cm to 1.5 cm from the GTV to determine the CTV; this estimation of the margin is largely empirical and mainly left to the discretion of the physician. In this study, the majority (94.7%) of patients with HCC had ME ≤ 3.5 mm, and the extent of ME depended on the tumor grade. Only 4 of 76 patients had ME > 4.0 mm beyond the GTV. The CTV margins of 0.2, 4.5, and 8.0 mm beyond the gross tumor would have been adequate to cover all the ME observed in this study for tumor Grades 1, 2, and 3, respectively, in all patients with HCC. Similarly, Wang et al retrospectively reviewed 300 slides from tumor samples of 149 patients with HCC using routine histopathologic preparations and recommended GTV-to-CTV expansions of 4 mm with 100% accuracy [23].

The level of serum AFP is associated with both survival and intrahepatic recurrence for HCC [29-32]. Montorsi et al. reported that the level of AFP is an independent predictor of disease recurrence in patients with HCC [31]. Imamura et al. revealed that a serum AFP level ≥32 ng/ml contributed to early intrahepatic recurrence of HCC after hepatectomy [32]. These findings were supported by the outcome of our study, in which a high prevalence of ME was noted in patients with an elevated AFP level. A previous study confirmed that the extent of microinvasion of HCC was correlated with high AFP levels [23]. However, elevated AFP levels were not associated with the distance of the ME in our study, this finding requires validation with larger patient numbers. Other studies suggest that serum AFP level probably reflects the degree of cellular differentiation, and thus, the extent of the tumor invasion [33-36]. Interestingly, the presence and extent of ME of prostatic carcinomas have also shown to correlate with increased PSA levels in patients with early stages of prostate cancer [17,18].

High tumor grades are associated with poor outcome for HCC patients. Shah et al. demonstrated that moderate (hazard ratio [HR], 3.0; 95% confidence interval [CI], 1.4-6.7) and poor (HR, 3.3; 95% CI, 1.3-8.3) tumor differentiation is an independent predictor of recurrence after resection of HCC [37]. Cucchetti et al. analyzed the prognostic factors of recurrence after resection of HCC; the results showed that poorly differentiated and undifferentiated tumors have a higher recurrence rate (54%) than well-differentiated and moderately differentiated tumors (25%; *P *= 0.015) [38]. Wayne et al. also demonstrated that tumor grade is a risk factor for HCC recurrence after resection [39]. Our data indicated that tumor grade was significantly associated with both the presence and extent of ME. Similarly, other studies revealed a significant correlation between the extent of extracapsular extension and higher Gleason scores in prostate cancer patients [17,18,40]. These data may partially explain why tumor recurrence was more frequent in patients with high-grade tumors than in those with low-grade tumors.

In this series, no correlation was observed between tumor size and extent of ME in HCC, which is consistent with the findings of other studies focusing on the extent of ME in metastatic lymph nodes and primary lung cancers [15,21]. These correlations of the extent of ME with the pathological features of the tumor and not the tumor size signify that ME may be related to the biological characteristics of the primary tumor. In contrast, a correlation between tumor size and the presence or extent of microinvasion was observed in a previous study of 149 patients with HCC [23]. Therefore, the correlations of the extent of ME with the tumor grade or the tumor size are needed to be further validated in the near future.

This is only a prospective study to evaluate microscopic extension using whole-mount preparations of HCC; however, our study has some potential limitations. First, the slides of specimens were only representative 2-dimensional sections of the resected tumor and may not really illustrate the ME in 3 dimensions. Some ME measurements may have been slightly underestimated. Second, sampling error was a possibility as there were a median of 5 slides examined per patient. Third, this study addressed histologic data, not radiographic. So even though ME was found beyond the histologically visible gross disease, this does not necessarily relate to the CTV and GTV, as used in radiation therapy, which are radiographically based definitions. Fourth, tissue shrinkage was not taken into consideration in this study. In a well-designed analysis of patients with prostate cancer, Schned et al. observed only 4.3% linear tissue shrinkage between the microscopic slide and the fresh specimen [41]. From a volumetric standpoint, it can be postulated that tissue shrinkage is lesser in HCC than in prostate cancer, owing to the presence of smooth muscles in the prostate gland and the absence of myofibroblastic cells in the liver parenchyma and neoplasms. Thus, it can be assumed that tissue shrinkage may be much smaller, perhaps even negligible.

## Conclusions

In summary, our clinicopathologic analysis indicated that the incidence of ME positively correlated with elevated AFP levels and high grades of tumor and that the extent of ME appears to be related only to the tumor grade. Although the optimal CTV of HCC remains undefined, it is reasonable to recommend CTV margins extending to ≤8 mm beyond the GTV for all tumor grades in patients with HCC.

## Competing interests

The authors declare that they have no competing interests.

## Authors' contributions

WW andYL designed the study, with assistance from XF. XF and WW analyzed the data. All authors helped to interpret the findings. WW wrote the manuscript, which was approved by all authors.

## References

[B1] Parkin DM Bray F Ferlay J Estimating the world cancer burden: GLOBOCAN 2000 Int J Cancer 2001 94 153 156 10.1002/ijc.1440 11668491

[B2] Jemal A Siegel R Ward E Cancer statistics, 2009 CA Cancer J Clin 2009 59 225 249 10.3322/caac.20006 19474385

[B3] Zhang SW Chen WQ Lei ZL A report of cancer incidence from 37 cancer registries in China, 2004 China Cancer 2008 17 909 912

[B4] Chen WQ Zhang SW Kong LZ Cancer mortality report of 34 cancer registries in China, 2004 China Cancer 2008 17 913 916

[B5] Llovet JM Burroughs A Bruix J Hepatocellular carcinoma Lancet 2003 362 1907 1917 10.1016/S0140-6736(03)14964-1 14667750

[B6] Llovet JM Updated treatment approach to hepatocellular carcinoma J Gastroenterol 2005 40 225 235 10.1007/s00535-005-1566-3 15830281

[B7] Choi TK Edward CS Fan ST Results of surgical resection for hepatocellular carcinoma Hepatogastroenterology 1990 37 172 175 1692802

[B8] Nagorney DM van Heerden JA Ilstrup DM Primary hepatic malignancy: surgical management and determinants of survival Surgery 1989 106 740 748 2799650

[B9] Hawkins MA Dawson LA Radiation therapy for hepatocellular carcinoma: from palliation to cure Cancer 2006 106 1653 1663 10.1002/cncr.21811 16541431

[B10] Fukumitsu N Sugahara S Nakayama H A prospective study of hypofractionated proton beam therapy for patients with hepatocellular carcinoma Int J Radiat Oncol Biol Phys 2009 74 831 836 19304408 10.1016/j.ijrobp.2008.10.073

[B11] Park W Lim DH Paik SW Local radiotherapy for patients with unresectable hepatocellular carcinoma Int J Radiat Oncol Biol Phys 2005 61 1143 1150 15752895 10.1016/j.ijrobp.2004.08.028

[B12] Mornex F Girard N Beziat C Feasibility and efficacy of high-dose three-dimensional radiotherapy in cirrhotic patients with small-size hepatocellular carcinoma non-eligible for curative therapies--mature results of the French phase II RTF-1 trial Int J Radiat Oncol Biol Phys 2006 66 1152 1158 17145534 10.1016/j.ijrobp.2006.06.015

[B13] Kim TH Kim DY Park JW Three-dimensional conformal radiotherapy of unresectable hepatocellular carcinoma patients for whom transcatheter arterial chemoembolization was ineffective or unsuitable Am J Clin Oncol 2006 29 568 575 10.1097/01.coc.0000239147.60196.11 17148993

[B14] Jenkins P Anjarwalla S Gilbert H Defining the clinical target volume for bladder cancer radiotherapy treatment planning Int J Radiat Oncol Biol Phys 2009 75 5 1379 84 19394154 10.1016/j.ijrobp.2009.01.063

[B15] Apisarnthanarax S Elliott DD El-Naggar AK Determining optimal clinical target volume margins in head-and neck cancer based on microscopic extracapsular extension of metastatic neck nodes Int J Radiat Oncol Biol Phys 2006 64 678 683 16243444 10.1016/j.ijrobp.2005.08.020

[B16] Yuan S Meng X Yu J Determining optimal clinical target volume margins on the basis of microscopic extracapsular extension of metastatic nodes in patients with non-small cell lung cancer Int J Radiat Oncol Biol Phys 2007 67 727 734 17293231 10.1016/j.ijrobp.2006.08.057

[B17] Chao KK Goldstein NS Yan D Clinicopathologic analysis of extracapsular extension in prostate cancer: should the clinical target volume be expanded posterolaterally to account for microscopic extension? Int J Radiat Oncol Biol Phys 2006 65 999 1007 16750320 10.1016/j.ijrobp.2006.02.039

[B18] Schwartz DJ Sengupta S Hillman DW Prediction of radial distance of extraprostatic extension from pretherapy factors Int J Radiat Oncol Biol Phys 2007 69 411 418 17869661 10.1016/j.ijrobp.2007.03.016

[B19] Teh BS Bastasch MD Wheeler TM IMRT for prostate cancer: defining target volume based on correlated pathologic volume of disease Int J Radiat Oncol Biol Phys 2003 56 184 191 12694837 10.1016/s0360-3016(03)00085-3

[B20] Grills IS Fitch DL Goldstein NS Clinicopathologic analysis of microscopic extension in lung adenocarcinoma: defining clinical target volume for radiotherapy Int J Radiat Oncol Biol Phys 2007 69 334 341 17570609 10.1016/j.ijrobp.2007.03.023

[B21] Giraud P Antoine M Larrouy A Evaluation of microscopic tumor extension in non-small-cell lung cancer for three-dimensional conformal radiotherapy planning Int J Radiat Oncol Biol Phys 2000 48 1015 1024 10.1016/S0360-3016(00)00750-1 11072158

[B22] Choo R Woo T Assaad D What is the microscopic tumor extent beyond clinically delineated gross tumor boundary in nonmelanoma skin cancers? Int J Radiat Oncol Biol Phys 2005 62 1096 1099 15990014 10.1016/j.ijrobp.2004.12.069

[B23] Wang MH Ji Y Zeng ZC Impact Factors for Microinvasion in Patients with Hepatocellular Carcinoma: Possible Application to the Definition of Clinical Tumor Volume Int J Radiat Oncol Biol Phys 2009 in press 19406586 10.1016/j.ijrobp.2009.01.057

[B24] Dawson LA McGinn CJ Normolle D Escalated focal liver radiation and concurrent hepatic artery fluorodeoxyuridine for unresectable intrahepatic malignancies J Clin Oncol 2000 18 2210 2218 10829040 10.1200/JCO.2000.18.11.2210

[B25] Park HC Seong J Han KH Dose-response relationship in local radiotherapy for hepatocellular carcinoma Int J Radiat Oncol Biol Phys 2002 54 150 155 10.1016/S0360-3016(02)03283-2 12182985

[B26] Liu MT Li SH Chu TC Three-dimensional conformal radiation therapy for unresectable hepatocellular carcinoma patients who had failed with or were unsuited for transcatheter arterial chemoembolization Jpn J Clin Oncol 2004 34 532 539 10.1093/jjco/hyh089 15466827

[B27] Ben-Josef E Normolle D Ensminger WD Phase II trial of high-dose conformal radiation therapy with concurrent hepatic artery floxuridine for unresectable intrahepatic malignancies J Clin Oncol 2005 23 8739 8747 10.1200/JCO.2005.01.5354 16314634

[B28] Seong J Park HC Han KH Clinical results and prognostic factors in radiotherapy for unresectable hepatocellular carcinoma: a retrospective study of 158 patients Int J Radiat Oncol Biol Phys 2003 55 329 336 12527045 10.1016/s0360-3016(02)03929-9

[B29] Carr BI Buch SC Kondragunta V Tumor and liver determinants of prognosis in unresectable hepatocellular carcinoma: A case cohort study J Gastroenterol Hepatol 2008 23 1259 1266 10.1111/j.1440-1746.2008.05487.x 18699979

[B30] Changchien CS Chen CL Yen YH Analysis of 6381 hepatocellular carcinoma patients in southern Taiwan: Prognostic features, treatment outcome, and survival J Gastroenterol 2008 43 159 170 10.1007/s00535-007-2134-9 18306990

[B31] Montorsi M Santambrogio R Bianchi P Survival and recurrences after hepatic resection or radiofrequency for hepatocellular carcinoma in cirrhotic patients: a multivariate analysis J Gastrointest Surg 2005 9 62 67 10.1016/j.gassur.2004.10.003 15623446

[B32] Imamura H Matsuyama Y Tanaka E Risk factors contributing to early and late phase intrahepatic recurrence of hepatocellular carcinoma after hepatectomy J Hepatol 2003 38 200 207 10.1016/S0168-8278(02)00360-4 12547409

[B33] Chevret S Trinchet JC Mathieu D A new prognostic classification for predicting survival in patients with hepatocellular carcinoma J Hepatol 1999 31 133 141 10.1016/S0168-8278(99)80173-1 10424293

[B34] Hanazachi K Kajikawa S Koide N Prognostic factors after hepatic resection for hepatocellular carcinoma with hepatitis C viral infection: Univariate and multivariate analysis Am J Gastroenterol 2001 96 1243 1250 10.1111/j.1572-0241.2001.03634.x 11316177

[B35] Matsumoto K Yoshimoto J Sugo H Relationship between the histological degrees of hepatitis and the postoperative recurrence of hepatocellular carcinoma in patients with hepatitis C Hepatol Res 2002 23 196 201 10.1016/S1386-6346(01)00180-2 12076715

[B36] Harrison LEH Koneru B Baramipour P Locoregional recurrences are frequent after radiofrequency ablation for hepatocellular carcinoma J Am Coll Surg 2003 197 759 764 10.1016/S1072-7515(03)00750-6 14585410

[B37] Shah S Cleary SP Wei MA Recurrence after liver resection for hepatocellular carcinoma: Risk factors, treatment, and outcomes Surgery 2007 141 330 339 10.1016/j.surg.2006.06.028 17349844

[B38] Cucchetti A Vivarelli M Piscaglia F Tumor doubling time predicts recurrence after surgery and describes the histological pattern of hepatocellular carcinoma on cirrhosis J Hepatol 2005 43 310 316 10.1016/j.jhep.2005.03.014 15970351

[B39] Wayne JD Lauwers GY Ikai I Preoperative predictors of survival after resection of small hepatocellular carcinomas Ann Surg 2002 235 722 731 10.1097/00000658-200205000-00015 11981219 PMC1422499

[B40] Wheeler TM Dillioglugil O Kattan MW Clinical and pathological significance of the level and extent of capsular invasion in clinical stage T1-2 prostate cancer Hum Pathol 1998 29 856 862 10.1016/S0046-8177(98)90457-9 9712429

[B41] Schned A Wheeler K Hodorowski C Tissue-shrinkage correction factor in the calculation of prostate cancer volume Am J Surg Pathol 1996 20 1501 1506 10.1097/00000478-199612000-00009 8944043

